# Step-to-Step Kinematic Validation between an Inertial Measurement Unit (IMU) 3D System, a Combined Laser+IMU System and Force Plates during a 50 M Sprint in a Cohort of Sprinters

**DOI:** 10.3390/s21196560

**Published:** 2021-09-30

**Authors:** Roland van den Tillaar, Ryu Nagahara, Sam Gleadhill, Pedro Jiménez-Reyes

**Affiliations:** 1 Department of Sports Sciences, Nord University, 7600 Levanger, Norway; 2 National Institute of Fitness and Sports in Kanoya, Kanoya 891-2393, Kagoshima, Japan; nagahara@nifs-k.ac.jp (R.N.); gleadhill@nifs-k.ac.jp (S.G.); 3 Centre for Sport Studies, Rey Juan Carlos University, 28943 Madrid, Spain; pedro.jimenezr@urjc.es

**Keywords:** xsens, musclelab, force plates, inertial sensors

## Abstract

The purpose was to compare step-by-step kinematics measured using force plates (criterion), an IMU only and a combined laser IMU system in well-trained sprinters. Fourteen male experienced sprinters performed a 50-m sprint. Step-by-step kinematics were measured by 50 force plates and compared with an IMU-3D motion capture system and a combined laser+IMU system attached to each foot. Results showed that step kinematics (step velocity, length, contact and flight times) were different when measured with the IMU-3D system, compared with force plates, while the laser+IMU system, showed in general the same kinematics as measured with force plates without a systematic bias. Based upon the findings it can be concluded that the laser+IMU system is as accurate in measuring step-by-step kinematics as the force plate system. At the moment, the IMU-3D system is only accurate in measuring stride patterns (temporal parameters); it is not accurate enough to measure step lengths (spatial) and velocities due to the inaccuracies in step length, especially at high velocities. It is suggested that this laser+IMU system is valid and accurate, which can be used easily in training and competition to obtain step-by step kinematics and give direct feedback of this information during training and competition.

## 1. Introduction

Sprinting performance is very important in many sports. The two main variables, step length and frequency, determine sprint performance. In general, taller athletes have longer step lengths and a lower frequency compared with shorter athletes who show the opposite [1]. To enhance sprinting performance, it is natural to enhance one of the two variables, while keeping the other constant [2]. The easiest way to measure mean step length and frequency is just by counting the number of steps over a certain distance and time, and then dividing the distance by the number of steps. However, during a sprint, step length and frequency are not constant and differ during the different phases of a sprint [3,4]. For athletes, coaches and scientists it can be important to know the step-by step kinematics to check how they respond to a stimulus (e.g., a que or training session/period).

There are several systems to measure step length and frequency, such as video, high speed cameras (Mero and Komi, 1985), force plates [1], photo/laser mat systems [5], and 3D systems (camera- and inertial measurement unit (IMU)-based) [6,7]. Force plates and 3D systems based upon several high-speed cameras have typically been considered the “gold standard” for measuring step characteristics with demonstrated errors/accuracy within mm [8,9]. However, force plates and these 3D systems are very expensive (several force plates and cameras) and require specialized expertise to analyze and thereby are unique to high performance laboratories. In contrast, wearable IMU systems may provide a practical, easy-to-use, portable and cheaper alternative to force platforms or 3D systems to measure step length and frequency, although it is unknown whether new/recent IMU systems and methodologies provide comparable accuracy.

IMUs can be used in several different ways. One of the possibilities is to measure movement patterns and specific step kinematics. However, the use of IMUs faces various challenges, especially the step length estimation errors and heading drift in walking, running and sprinting [10]. Recent studies have found that with IMU it is possible to detect step patterns during walking, running and sprinting [10,11]. Horsley et al. [12] showed in a recent review that placement of IMUs on the foot, tibia and lumbar spine was suitable to derive valid and reliable stride data (step length and frequency, flight and contact times) during running with velocity ranges between 3.3–4.3 m/s. de Ruiter and van Dieën [11] showed with well-trained sprinters that step length and over all steps during maximal sprint acceleration was accurately measured (root mean square error = 5.7 cm, bias ± limits of agreement = −0.15 ± 11 cm) when IMUs were combined with timing gates over 60m. However, to the best of our knowledge, no study has investigated step-by-step kinematics, including contact and flight times measured with IMUs in maximal sprinting and validated this.

A system (Laser Speed) as part of the MUSCLELAB system (Ergotest Technology AS, Langesund, Norway) was introduced that combines a laser with IMUs on each foot that automatically detects step-by-step kinematics [7]. Full 3D IMU-based systems are commercially available, in which Xsens MVN Analyze (Xsens Technologies B.V., Enschede, The Netherlands) is known as one of the pioneers and shows reliable data in walking [13]. However, neither system has been validated against a gold standard like force plates during sprinting. Therefore, the aim of the present study was to compare measurements of step-by-step kinematics of maximal sprinting between force plates (gold standard), an IMU-3D system and a combined laser+IMU system with experienced sprinters.

## 2. Materials and Methods

### 2.1. Participants

Fourteen well-trained male sprinters (age: 20.8 ± 1.4 years, mass: 68.1 ± 6.2 kg, height: 1.75 ± 0.06 m, 100 m time: 11.24 ± 0.33 s) participated in this study. Each participant was informed of the testing procedures and possible risks and written consent was obtained prior to the study. The study was conducted with the approval of the local ethical committee and conformed to the latest revision of the Declaration of Helsinki.

### 2.2. Procedure

**Figure 1:**
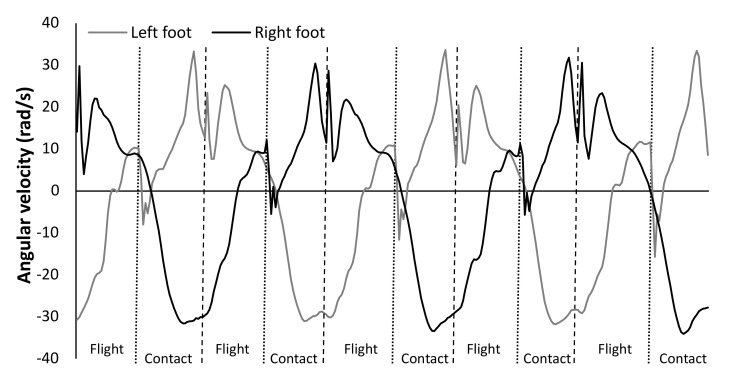
A typical example of five steps of the raw plantar flexion pattern measured with an IMU (Xsens system) attached on the left and right foot with corresponding contact and flight time phases.

After an individualized warm-up, each participant performed a 50-m sprint with maximal effort from a start block, wearing spikes. Each participant put on the Xsens lycra suit (Xsens Technologies B.V., Enschede, The Netherlands), which consisted of 17 IMUs (240 Hz) attached to the athletes’ spikes, shanks, thighs, pelvis, sternum, head, upper arms, forearms, and hands (https://www.xsens.com/products/mvn-analyze) (accessed on 10 November 2020). To construct a rigid body 23-link MVN model for kinematic analysis, height of the participants and foot length were input into the software (MVN Analyze 2019, Xsens, Netherlands). The IMUs for the feet were fixed by tape over the spikes. Upright posture (N-pose) and walking movements were then recorded for model calibration. The software automatically calculated joint angles, joint velocities, and center of mass (COM) over time (https://www.xsens.com/products/mvn-analyze) (accessed on 10 November 2020). To identify contact and flight times of each step, the pattern of angular velocity of ankle plantar flexion/extension was used, which coincided with the contact and flight times (ICC = 0.94) measured with an infrared contact mat over 30 m (Ergotest Technology AS, Langesund, Norway) determined in an unpublished pilot study (Figure 1) and by visual confirmation using the MVN Analyse program. Step length was based on the distance of the COM travelled for each step (contact and flight times). Step velocity and frequency were calculated by respectively the step length and 1 divided by the total contact and flight times of each step.

The combined laser+IMU system (Laser Speed) as part of the MUSCLELAB system (Ergotest Technology AS, Langesund, Norway) recorded distance over time continuously during each attempt using a CMP3 Distance Sensor laser gun (Noptel Oy, Oulu, Finland), sampling at 2.56 KHz. Throughout each sprint contact and flight times (identified in the same way as in the IMU-3D system) together with step length (distance between two adjacent contact times measured with laser) and frequency (1/contact + flight time step) were automatically detected by the software using wireless, 9 degrees of freedom, IMUs integrated with a 3-axis gyroscope attached on top of the shoelaces of the spikes of each foot directly up the IMUs of the 3D-IMU system. Sampling rate of the IMU was 1000 Hz with maximal measuring range of 2000° s^−1^ ± 3% (Ergotest Technology AS, Langesund, Norway). All recordings of the IMUs and the laser were synchronized with the MUSCLAB v10.57 (Ergotest Technology AS, Langesund, Norway).

As the gold standard for step-by-step kinematics, 54 interconnected force platforms (dimensions measure 900 mm × 1000 mm) were used from 1.5 m behind the start line to the 50 m mark (TF-90100, TF-3055, TF-32120, Tec Gihan, Uji, Japan) sampling at 1000 Hz. A digital 50 Hz low-pass, fourth-order Butterworth filter was applied to raw data. Foot strike and toe-off were identified using vertical ground reaction force by exceeding or falling beneath a 20 N threshold. Step duration was the time to complete each step and step frequency was the inverse of step duration. Contact time was the duration of ground contact (foot strike to toe-off) and flight time was the duration from toe-off to foot strike. Centre of pressure was calculated according to previous research [8] and the step-to-step center of pressure were the mean ground contact for 0.01 s during the middle of each steps’ contact phase. Step length was the anterior distance between step-to-step centers of pressure instant/location. The velocity was the product of step length and step frequency.

To compare the kinematics between the three different systems, 24 steps, from the first step touch down on which all three systems were synchronized, were used, since all participants performed at least 24 steps.

### 2.3. Statistical Analysis

Normality was tested using the Shapiro-Wilks test. To compare step kinematics, a 3 (measuring systems: force plates, laser+IMU, IMU-3D system) * 24 (steps) analysis of variance (ANOVA) with repeated measures was performed. A post hoc test was conducted with a Holm-Bonferroni correction to identify where the eventual differences in step kinematics occurred. If the sphericity assumption was violated, *p*-values of the Greenhouse-Geisser adjustment were reported. Furthermore, Bland–Altman limits of agreement method was used to identify potential systematic bias, which were reported through mean-bias and standard deviations. Pearson’s correlations were conducted to investigate if the eventual differences between measurement devices changed over the steps. The criteria to interpret the strength of the r coefficients were as follows: trivial (<0.1), small (0.1–0.3), moderate (0.3–0.5), high (0.5–0.7), very high (0.7–0.9), or practically perfect (>0.9) [14]. All results are presented as mean ± standard deviation (SD), and the alpha level was set at *p* < 0.05. The effect size for ANOVA was evaluated as η2 (Eta partial squared) where 0.01 < η^2^ < 0.06 constitutes a small effect, 0.06 < η^2^ < 0.14 constitutes a medium effect, and η^2^ > 0.14 constitutes a large effect [15]. Statistical analyses were performed in SPSS version 25.0 (SPSS, Inc., Chicago, IL, USA).

## 3. Results

**Figure 2:**
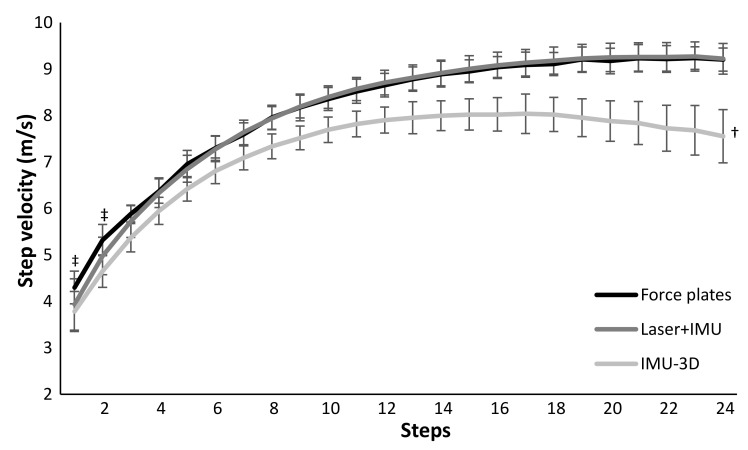
Mean (±SD) step-by-step velocity averaged over all participants during 50 m sprints measured with force plates, laser+IMU and IMU-3D based systems. † indicates a significant difference for each step for this condition with the force plate condition on a *p* < 0.05 level. ‡ indicates a significant difference for this step for laser+IMU system with the force plate condition on a *p* < 0.05 level.

**Figure 3:**
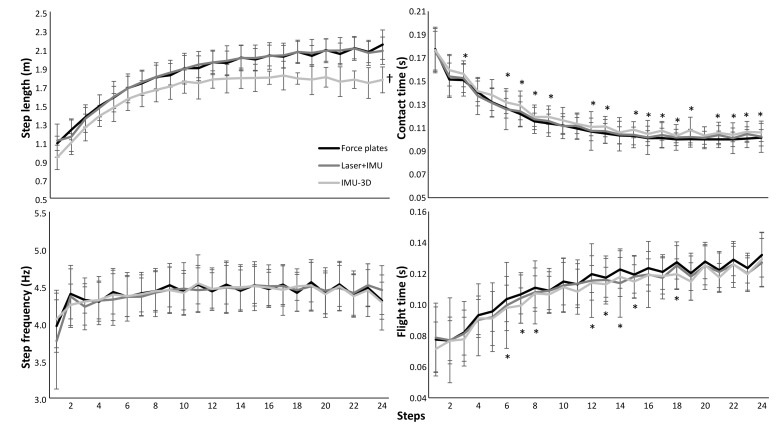
Mean (±SD) step-by-step step length, frequency, contact and flight time averaged over all participants during 50 m sprints measured with force plates, laser+IMU and IMU-3D based systems. † indicates a significant difference for each step for this condition with the force plate condition on a *p* < 0.05 level. * indicates a significant difference between the IMU-3D system with the force plate system for this step on a *p* < 0.05 level.

A significant effect of steps was found for all five kinematic variables (F ≥ 6.3, *p* ≤ 0.001, η^2^ ≥ 0.33). Also, a significant effect between the three measurement systems was found for step velocity (F = 76, *p* ≤ 0.001, η^2^ = 0.86), step length (F = 34, *p* ≤ 0.001, η^2^ = 0.72) and contact times (F = 11, *p* ≤ 0.001, η^2^ = 0.46) and a significant step*system interaction effect of these three kinematical variables was found (F ≥ 1.4, p ≤ 0.049, η^2^ ≥ 0.10). For step frequency and flight time no significant measurement system or interaction effects were found (F ≤ 2.3, *p* ≥ 0.134, η^2^ ≤ 0.15). Post hoc comparison showed that step velocity and step length measured with the IMU-3D system were lower in each step and followed another development trajectory over the 24 steps compared to that measured with force plates (Figure 2 and Figure 3). In addition, contact times, in most steps, were longer (16 steps) and flight times were shorter (9 steps) when measured with the IMU-3D system compared to force plates (Figure 3). There were no significant differences found between the laser+IMU system and the force plate system for any of the kinematics (Figure 2 and Figure 3), except when evaluating per step; in the first two steps, a significant lower step velocity was found in the laser+IMU system compared with the force plates.

**Figure 4:**
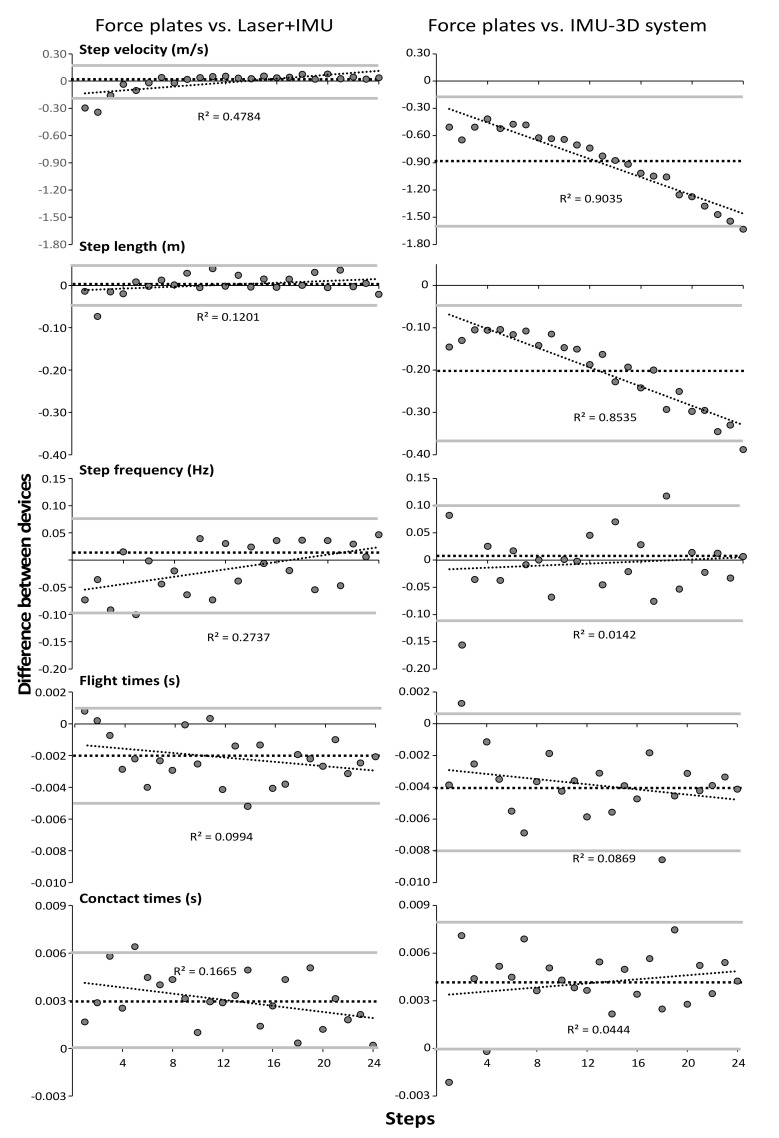
Bland-Altman plots and linear correlations between force plate, laser+IMU and IMU-3D systems for each kinematics parameter. Dashed lines indicate a systematic bias between the measuring devices (positive values mean higher values obtained with the IMU systems than force plates). The grey lines represent 95% confidence intervals.

A significant systematic bias over all steps in step velocity (−0.88 m/s) and step length (−0.20 m) between the IMU-3D system and force plates was found in which the step velocity and step length were measured slower and shorter in the IMU-3D system compared with force plates (Figure 4). In addition, the average contact times over all steps were significantly longer (0.003 and 0.004 s) and flight times were shorter (−0.002 and −0.004 s) when measured with the laser+IMU and IMU-3D system compared to force plates (Figure 4). However, when analyzing per step, this was mostly only shown in the IMU-3D system compared with force plates (Figure 3). Furthermore, some significant, moderate to nearly perfect correlations were found between steps and difference in measurement between the systems with force plates (Figure 4). Practically perfect, negative correlations were found in step velocity (r = −0.95) and length (−0.92), and trivial for the other parameters (−0.29 < r < 0.21) between IMU-3D and force plate measurements, while significant, high, positive correlations were found in step velocity (r = 0.69) and step frequency (0.53), moderate in step length (0.35) and contact times (−0.36), and trivial for flight time (−0.23) between laser+IMU and force plate measurements (Figure 4).

## 4. Discussion

The aim of the study was to compare the step-by step kinematics between three systems. The main findings were that step kinematics were different when measured with the IMU-3D system compared with force plates, while the laser+IMU system showed, in general, the same kinematics as measured by force plates, without systematic bias.

The IMU-3D system measured lower step velocity in each step (−0.88 m/s) compared with force plates (Figure 2 and Figure 4), which is mainly caused by the shorter measured step length in each step (−0.20 m). This difference increased over the sprinting distance as the step velocity increased, as shown by the very strong correlation over the steps in velocity and step length (Figure 4). A possible reason for the differences in step length, and thereby velocity, is the calibration of the IMU-3D system. The system calibrates by participants standing, walking forward around 10m, and returning to the start position. This results in only small accelerations and spatial displacements of the IMUs. During maximal sprinting these displacements are much larger than in the calibration; therefore, small inaccuracies can cause large differences in step velocity. For example, a 0.3 m shorter step length during a maximal sprint (1.7 m vs. 2 m), results in the same total step time (flight + contact time ≈ 0.23 s) with a difference of ~1.4 m/s (7.4 vs. 8.7 m/s), as shown in Figure 4. With the laser+IMU system, significantly lower step velocity was found in the first two steps, which was caused by shorter step length, due to the detection distance of the measuring tool. The force plate measures step length accurately based on the center of pressure, whereas the IMUs of the laser+IMU system only detects whether the foot is on the ground; distance is measured by a laser that is pointed at the back of the subject when the foot is in contact with the ground. The difference in distance between the two following ground contacts is recorded as step length. In the first few steps, athletes lean forward [16]. Since foot placement is behind the center of mass and the trunk is leaning forwards, the laser records the displacement of the lumbar point instead of the center of mass [17]. Thereby, differences are observed between the measurements produced by the two measurement systems, which only occur in the first two steps [5].

When analyzing the error per step, a systematic bias was found for both IMU systems with force plates in which IMU systems measured longer contact times (0.003 and 0.004 s) and shorter flight times (−0.002 and −0.004 s) when evaluating overall steps. This error is mainly based upon the identification of touch down and toe off events with the IMU and sampling rate. Touch down and toe off events were identified in a pilot study, in which an infrared mat was used which makes a grid 3mm above the surface, and compared with IMU patterns. When the grid is broken, it identifies that the foot is on the ground. Since the grid is a few mm above the surface the contact time is measured longer (a few msec) and flight time is shorter than with the force plates. The IMU-3D system only measured with 240 Hz, which means that it sampled every 0.0042 s. This means that the difference with the force plates is just one sample duration difference, on average, in contact and flight time. As no differences were found in step frequency between the three systems, it is just the identification of the touch down or toe off that probably needs to change just one sample duration in both IMU systems to produce valid contact and flight time readings compared with the force plates. Therefore, it is suggested, based upon the present findings, to correct the toe off event by one sample duration earlier (in IMU-3D system). Thus, at the first peak, angular plantar flexion velocity, instead of the dip right after the peak velocity (Figure 1). This would make the contact and flight times comparable with the gold standard.

In summary, it can be concluded that the laser+IMU system is a valid system compared with force plates, and that the IMU-3D system is only valid for temporal readings, and not spatial readings, especially when the step velocity increases during sprinting. Therefore, the laser+IMU system is a system that now could be trusted to be used practically for coaching/research and is easy to use in training, as it is portable, easy to set up (two IMUs on the spikes and a laser connected to small box to a laptop) and automatically calculates step-by-step kinematics after every sprint. Thereby, the laser+IMU system gives the possibility of immediate feedback to athletes and their coaches. The IMU-3D system has more limitations, as athletes have to wear a Lycra suit, which could be too warm in a hot climate and too time consuming to put on the system. Furthermore, the system has no automatic detection of the steps and the spatial parameters are not accurate enough.

In summary, based upon the findings of the present study it can be concluded that the laser+IMU system is as accurate in measuring step-by-step kinematics as the force plate system, while the IMU-3D system is good in measuring patterns (temporal parameters, like contact and flight times), but is not accurate enough to measure step lengths and velocities (spatial parameters) during sprinting. It is suggested that this laser+IMU system is a good and accurate system, which can easily be used in training and competition to obtain step-by step kinematics and give direct feedback of this information to athletes during training and competition.

## Data Availability

The data presented in this study are available on request from the corresponding author. The data are not publicly available due to national laws of the Norwegian and Japanese government on privacy.
